# Traditional Cattle Grazing in a Mosaic Alkali Landscape: Effects on Grassland Biodiversity along a Moisture Gradient

**DOI:** 10.1371/journal.pone.0097095

**Published:** 2014-05-08

**Authors:** Péter Török, Orsolya Valkó, Balázs Deák, András Kelemen, Béla Tóthmérész

**Affiliations:** 1 MTA-DE Biodiversity and Ecosystem Services Research Group, Debrecen, Hungary; 2 Department of Ecology, University of Debrecen, Debrecen, Hungary; Stockholm University, Sweden

## Abstract

Extensively managed pastures are of crucial importance in sustaining biodiversity both in local- and landscape-level. Thus, re-introduction of traditional grazing management is a crucial issue in grassland conservation actions worldwide. Traditional grazing with robust cattle breeds in low stocking rates is considered to be especially useful to mimic natural grazing regimes, but well documented case-studies are surprisingly rare on this topic. Our goal was to evaluate the effectiveness of traditional Hungarian Grey cattle grazing as a conservation action in a mosaic alkali landscape. We asked the following questions: (i) How does cattle grazing affect species composition and diversity of the grasslands? (ii) What are the effects of grazing on short-lived and perennial noxious species? (iii) Are there distinct effects of grazing in dry-, mesophilous- and wet grassland types? Vegetation of fenced and grazed plots in a 200-ha sized habitat complex (secondary dry grasslands and pristine mesophilous- and wet alkali grasslands) was sampled from 2006–2009 in East-Hungary. We found higher diversity scores in grazed plots compared to fenced ones in mesophilous- and wet grasslands. Higher cover of noxious species was typical in fenced plots compared to their grazed counterparts in the last year in every studied grassland type. We found an increasing effect of grazing from the dry- towards the wet grassland types. The year-to-year differences also followed similar pattern: the site-dependent effects were the lowest in the dry grassland and an increasing effect was detected along the moisture gradient. We found that extensive Hungarian Grey cattle grazing is an effective tool to suppress noxious species and to create a mosaic vegetation structure, which enables to maintain high species richness in the landscape. Hungarian Grey cattle can feed in open habitats along long moisture gradient, thus in highly mosaic landscapes this breed can be the most suitable livestock type.

## Introduction

Conservation and restoration of grassland biodiversity is a hot topic of ecological research and nature conservation practice [1]. Extensively managed pastures are of crucial importance for sustaining grassland biodiversity across Europe [2], [3]. Unfortunately, most of Europe’s former extensive pastures became intensively used or were abandoned [2]. The main reasons for intensification are to increase biomass production for forage and for bioenergy [4]. Abandonment occurs mostly on low production grasslands where former management regimes are not profitable any more [5]. Both phenomena led to unfavourable changes in species composition, loss of biodiversity and important ecosystem functions and services (biological control, pollination or seed dispersal) [6], [7]. Conservation of grassland biodiversity is especially important in agricultural landscapes, where extensively managed grasslands act as refuge for many threatened plant and animal species and have a crucial role in increasing landscape-scale biodiversity [8], [9].

Re-introduction and/or preservation of traditional management, especially low-intensity grazing, became an important issue in grassland conservation and management in Europe [10], [11], [12]. The importance of extensive grassland management by grazing was also highly rated in Agri-Environmental Schemes and substantial support of these practices was assigned in the form of subsidy payments [3], [13], [14]. Traditional low-intensity grazing is considered to be important in (i) sustaining biodiversity, (ii) facilitation of the immigration and establishment of desirable species and in (iii) the suppression of noxious ones. Compared to the first two issues proportionally less attention was given to the latter one (but see [15], [16]).

Cattle grazing is considered to be suitable for sustaining grassland biodiversity, because of its lower selectivity compared to sheep or horse grazing [16], [17]. However, the effects of cattle grazing strongly depend on the cattle breed and the duration and intensity of grazing [3]. Traditional cattle grazing systems with robust cattle breeds (e.g. Heck cattle or Hungarian Grey cattle) in low stocking rates are considered to be proper to mimic natural grazing regimes in grasslands [18]. Thus, this management type is increasingly introduced in nature conservation and restoration practice in many parts of Europe [19].

**Figure 1:**
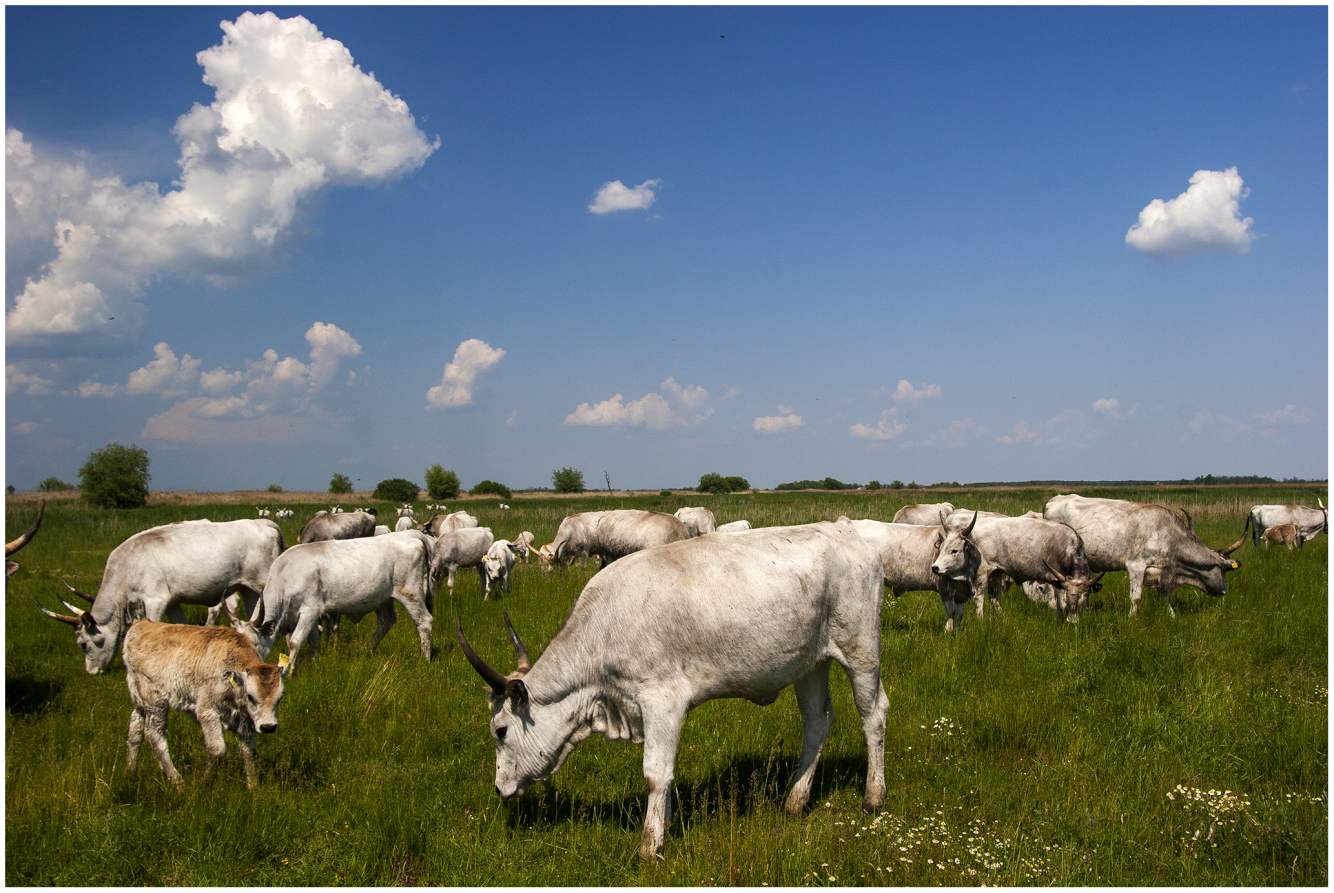
Hungarian Grey cattle grazing in the study area. Photo by Balázs Deák.

Alkali landscapes are traditionally managed by low intensity cattle and/or sheep grazing [20]. In Central-Europe extensive pastures were grazed by Hungarian Grey cattle, which is a traditional beef cattle breed in the Carpathian-basin and neighbouring countries from about the 13^th^ century onwards [21] (Figure 1). Socio-economical changes during the socialist era, collectivisation and the switch from traditional to industrial food production resulted in a decrease in overall livestock numbers and a considerable decrease in traditional herding [20], [22]. All of these negative changes resulted in a large-scale cessation of traditional grazing, especially in the low productivity alkali landscapes [23], [24].

In the last 25 years, several attempts were initiated to recover former traditional management by grazing and accordingly Hungarian Grey cattle grazing was reintroduced [24]. However, only a few case studies are available publishing evidences on the effects of Hungarian Grey cattle grazing on the vegetation composition [25]. As being a promising tool for nature conservation and restoration projects it is crucial to have comprehensive evidence-based knowledge on the effects of Hungarian Grey cattle grazing on species composition of the vegetation. This knowledge is essential for the planning and evaluation of conservation and restoration projects. In this paper we evaluated the effectiveness of traditional Hungarian Grey cattle grazing in suppressing noxious species in three grassland types of a mosaic alkali landscape. We asked the following questions: (i) How does cattle grazing affect species composition and diversity of the three grassland types? (ii) What are the effects of grazing on short-lived and perennial noxious species? (iii) Are there distinct effects of grazing for dry-, mesophilous- and wet grassland types?

## Materials and Methods

### Sampling Setup

The study area is in the region of the ‘Egyek-Pusztakócsi mocsarak’ marshland-grassland system (N 47° 33′, E 20° 55′) which forms an integral part of the Hortobágy National Park, East-Hungary. A landscape-scale grassland restoration project was initiated in 2004 funded by the EU LIFE program [26]. In this project 760 hectares of former croplands were re-grassed using low diversity seed mixtures of native grasses [27]. Besides grassland restoration, the project aimed at reintroduce traditional grazing regimes by Hungarian Grey cattle and sheep in several parts of the marshland-grassland system. In the present study we report the short-term effects of the newly introduced grazing of the Hungarian Grey cattle on the composition of three grassland types. Vegetation of a 200 ha sized habitat complex was sampled, which consisted of a large secondary dry grassland (spontaneously recovered dry loess grassland in a 10-year-old former lucerne field) at the highest elevations, bordered by pristine mesophilous- and wet alkali grasslands at the lower elevations. Three independent transects (in at least 200-m-distance from each other) were selected from the secondary dry grassland towards the lower-laying mesophilous and wet grasslands. Along each transect one sampling site (in the following ‘site’) with two sampling plots (5×5-m-sized each) per grassland type were randomly placed. One of them was fenced (control) and the other one unfenced (grazed plot). Within the sampling plots there were four 1×1m-sized permanent subplots, systematically placed within the plot area in one meter distance both from neighbouring subplots and plot margin. From 2006 to 2009 the percentage cover of vascular plant species was recorded in early July (2006– before the introduction of grazing, and 2007–2009 after the yearly grazing started). During the study the whole area was grazed by Hungarian Grey cattle (from 2006 onwards; in 2006 from September till November, in the following years from early April till late October-November in one cattle per hectare grazing density).

### Data Processing

Species were considered as ‘noxious’ using [28], and we refined the categorization based on personal expertise of the authors and using the information listed in the Appendix of [29]. The complete list of noxious species is provided in Table S1. We calculated the cover-weighted relative ecological indicator values for soil moisture (WB) based on [30] and adapted to the local environmental conditions by [31]. To display the compositional diversity of vegetation Shannon diversity was calculated. Using the means of maximum-minimum plant heights reported in the identification book for the Hungarian flora [32] cover-weighted specific plant heights were calculated for each subplot. DCA ordination was used to assess the temporal changes in the composition of the three studied grassland types; it was calculated using CANOCO 4.5 program package [33]. In statistical calculations percentage cover scores were standardised with summarised total cover scores. Treatments were compared using three-factorial repeated measures GLM where ‘year’ was included as repeated measures factor, and ‘management’ (fenced vs. grazed) and ‘site’ as fixed factors. All univariate statistics were calculated using SPSS program package.

### Ethics Statement

The authors state that no authority permission was needed for their study, as the study did not affect any endangered or protected species, and was carried out with non-destructive methods for the habitats and the environment. The landowner of the area was the Hortobágy National Park Directorate, who approved the authors to access the area and carry out the research. The study sites were located at the N 47° 33′ 25.06″, E 20° 55′ 27.18″ coordinates.

## Results

### Species Composition and Diversity

Altogether 124 species were detected during the study, 84 species in secondary dry grasslands, 76 species in mesophilous grasslands and 69 species in wet grasslands. The highest diversity scores were typical in all years and almost all plots in secondary dry grasslands no significant effect of management was detected on species richness and Shannon diversity (Table 1, Table S2). In mesophilous- and wet grasslands both species richness and Shannon diversity were significantly affected by the management; generally higher species richness and Shannon diversity scores were typical after three years of management in grazed plots (Table 1 and Table S2). Except of the wet grasslands both species richness and Shannon diversity were significantly affected by the site. Generally, higher specific plant heights were significantly affected by management; lower scores were typical in fenced plots especially for the last two years of the study in all type of grasslands (Table 1, Table S2), due to the increase in cover of perennial short grasses (e.g. *Festuca pseudovina* and *Poa angustifolia*) and short rosette-forming and/or creeping species (e.g. *Plantago lanceolata*, *P. major*, *P. media*, *Taraxacum officinale*, *Trifolium repens* and *Polygonum aviculare*) in grazed plots in all grassland types.

**Table 1:** Effects of ‘year’, ‘management’ and ‘site’ on the vegetation characteristics in dry-, mesophilous- and wet grasslands.

Secondarydry grassland	Year		Manag.		Site		Year×Manag.		Year×Site		Manag.×Site		Year× Manag.×Site	
	F	*p*	F	*p*	F	*p*	F	*p*	F	*p*	F	*p*	F	*p*
Shannon diversity	2.954	0.065	0.001	0.997	3.941	**0.038**	2.204	0.127	1.033	0.423	1.848	0.186	0.779	0.593
Species richness	1.570	0.236	1.292	0.271	16.545	**<0.001**	0.764	0.531	3.116	**0.016**	0.771	0.477	0.562	0.757
Specific height	1.890	0.172	9.744	**0.006**	39.320	**<0.001**	0.989	0.423	2.839	**0.025**	0.257	0.776	0.660	0.682
Soil moisture (WB)	18.527	**<0.001**	0.415	0.528	43.100	**<0.001**	0.884	0.565	1.957	0.102	7.134	**0.005**	1.073	0.399
Noxious perennials	18.74	**<0.001**	1.661	0.214	22.428	**<0.001**	4.402	**0.019**	2.182	0.071	1.280	0.302	2.948	**0.021**
Noxious short-lived	0.204	0.892	27.340	**<0.001**	32.757	**<0.001**	2.916	0.066	2.262	0.062	6.537	**0.007**	3.277	**0.013**
**Mesophilous**	**Year**		**Manag.**		**Site**		**Year×Manag.**		**Year×Site**		**Manag.×Site**		**Year×Manag.×Site**	
**grassland**	**F**	***p***	**F**	***p***	**F**	***p***	**F**	***p***	**F**	***p***	**F**	***p***	**F**	***p***
Shannon diversity	1.397	0.280	11.770	**0.003**	31.966	**<0.001**	6.565	**0.004**	5.819	**<0.001**	2.557	0.105	6.982	**<0.001**
Species richness	1.019	0.410	25.070	**<0.001**	77.881	**<0.001**	1.146	0.361	5.355	**0.001**	2.148	0.146	1.864	0.118
Specific height	22.802	**<0.001**	19.998	**<0.001**	8.773	**0.002**	19.753	**<0.001**	7.525	**<0.001**	7.355	**0.005**	4.424	**0.002**
Soil moisture (WB)	1.185	0.347	0.777	0.390	15.396	**<0.001**	4.581	**0.017**	13.930	**<0.001**	9.892	**0.001**	2.238	0.065
Noxious perennials	5.892	**0.007**	2.945	0.103	46.027	**<0.001**	12.859	**<0.001**	1.581	0.185	1.520	0.246	2.505	**0.042**
Noxious short-lived	24.864	**<0.001**	3.156	0.093	57.707	**<0.001**	1.111	0.374	6.707	**<0.001**	2.867	0.083	0.979	0.456
**Wet**	**Year**		**Manag.**		**Site**		**Year×Manag.**		**Year×Site**		**Manag.×Site**		**Year×Manag.×Site**	
**Grassland**	**F**	***p***	**F**	***p***	**F**	***p***	**F**	***p***	**F**	***p***	**F**	***p***	**F**	***p***
Shannon diversity	14.501	**<0.001**	36.276	**<0.001**	1.914	0.176	2.697	0.081	5.360	**0.001**	42.442	**<0.001**	3.825	**0.005**
Species richness	25.564	**<0.001**	21.378	**<0.001**	4.652	**0.024**	6.019	**0.006**	4.973	**0.001**	13.020	**<0.001**	3.804	**0.006**
Specific height	112.489	**<0.001**	128.359	**<0.001**	25.887	**<0.001**	48.890	**<0.001**	4.394	**0.002**	7.610	**0.004**	5.592	**<0.001**
Soil moisture (WB)	37.141	**<0.001**	71.685	**<0.001**	6.372	**0.008**	10.982	**<0.001**	6.591	**<0.001**	4.101	**0.034**	2.424	**0.048**
Noxious perennials	14.842	**<0.001**	49.440	**<0.001**	24.770	**<0.001**	19.909	**<0.001**	1.834	0.124	8.576	**0.002**	3.645	**0.007**
Noxious short-lived	16.147	**<0.001**	0.511	0.484	1.747	0.203	5.481	**0.009**	2.250	0.064	1.111	0.351	2.475	**0.044**

In the three-factorial repeated measures GLM ‘year’ was included as repeated measures factor, and ‘management’ (fenced vs. grazed plots) and ‘site’ as fixed factors. Significant effects are marked with boldface. For ‘noxious perennials’ and ‘noxious short-lived’ species the cover scores were tested. For ‘WB’ and ‘specific height’ cover weighted scores were calculated and tested. See the detailed mean scores in Table S2.

The cover based soil moisture scores (WB) were higher in every fenced than in grazed plot in wet grasslands in the last year of the study. No such clear trends were found in the other two grassland types (Table 1, Table S2).

### Noxious Species

We found in total 47 noxious species (10 perennials and 37 short-lived species) in the three grassland types (see List of noxious species in Table S1). Contrasting results were found concerning the cover of noxious perennials in the three grassland types. In secondary dry grasslands higher cover scores of noxious species were typical in fenced plots compared to the grazed ones (Table S2); but the proportion of short-lived and perennial noxious species was highly variable between sites mainly due to the uneven pattern detected in the cover of *Elymus repens*, *Taraxacum officinale* and *Convolvulus arvensis*. Thus, significant effect of management was detected only for the noxious short-lived group (Table 1). Out of the noxious perennials *Calamagrostis epigeios* was exclusively found in grazed plots in the last year of the study.

**Figure 2:**
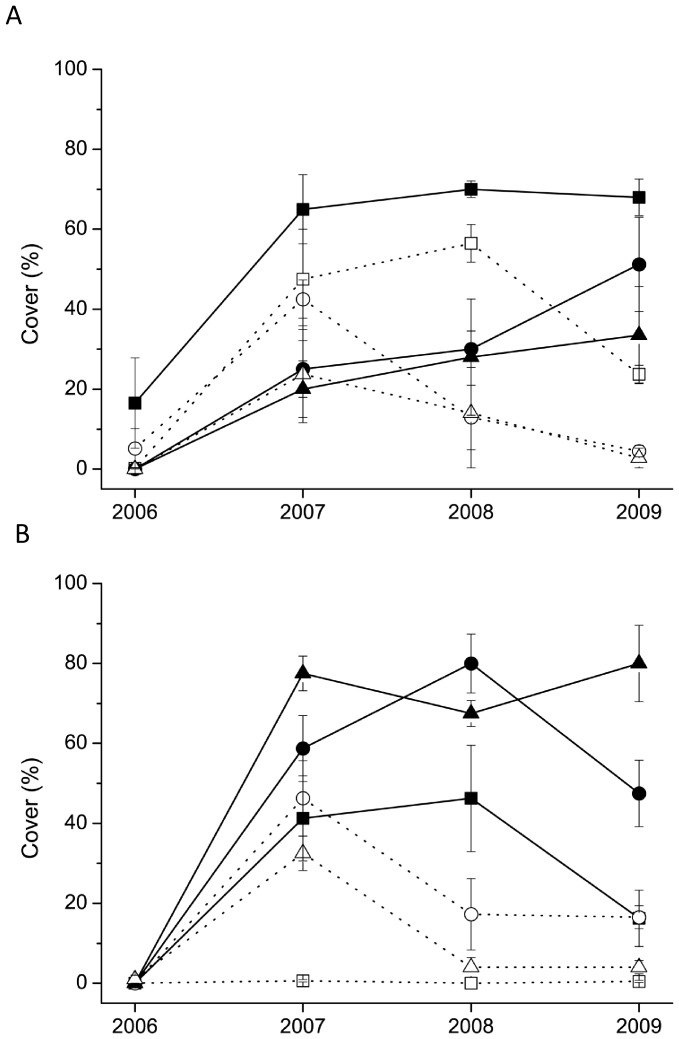
Cover scores of noxious species. Cover scores (mean±SE) of (**A**) *Elymus repens* in mesophilous grasslands and (**B**) *Phragmites communis* in wet grasslands in the four study years. Empty symbols with dotted line denote the grazed, full symbols with straight line denote the fenced plots. Rectangles are for Site 1, circles for Site 2, while triangles for Site 3.

Both in mesophilous and wet grasslands generally higher cover of noxious perennials were recorded in fenced plots compared to their grazed counterparts in the last year (Table S2). A significant effect of management on the cover of noxious perennials was detected both in mesophilous and wet grasslands (Table 1). In mesophilous grasslands *Elymus repens* was suppressed by grazing (Figure 2A), in the last year of the study almost five- to ten-times higher cover scores were typical in fenced plots. In wet grasslands this suppressive effect was the most feasible for *Phragmites communis*, three- to twenty-times higher scores were typical in fenced plots compared to the grazed ones (Figure 2B).

Noxious short-lived species were found in considerable cover only in secondary dry grasslands. A significant effect of management was detected only in secondary dry grasslands, where higher cover scores of noxious short-lived species was found in fenced plots than in the grazed ones (Table 1, Table S2). The most frequent noxious short-lived species, such as *Conyza canadensis, Matricaria inodora, Melandrium album, Picris hieracioides, Polygonum aviculare* and also the thistle *Carduus acanthoides* were suppressed by grazing.

### Effect of Grazing in Various Grassland Types

We found pronounced differences in the reaction of the grassland types to grazing depending on the moisture. From the dry towards the wet grassland types an increasing effect of grazing was found: out of the six studied vegetation characteristics in the secondary dry grasslands two characteristics (specific plant height, cover of noxious short-lived species), in the mesophilous grasslands three characteristics (Shannon diversity, species richness, specific plant height), in wet grasslands five characteristics (all, in exception of the cover of noxious short-lived species) were significantly affected. The year-to-year differences also followed a similar pattern: their effects were the lowest in the dry grassland type and an increasing effect was detected along the moisture gradient from the dry to the wet grassland type (Table 1).

**Figure 3:**
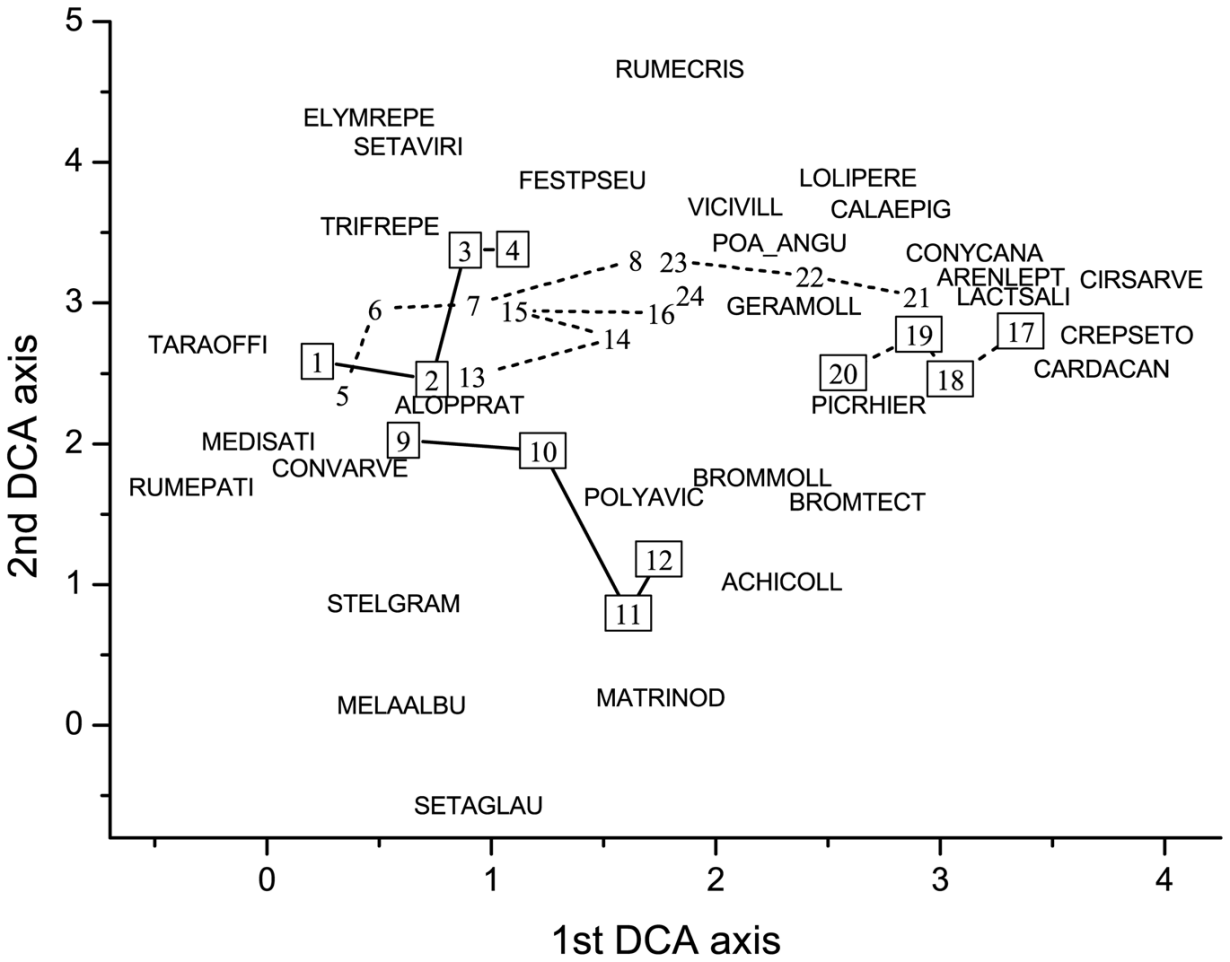
Vegetation changes in the secondary dry grasslands in the four years of the study. DCA ordination based on cover scores (gradient lengths, cumulative percentage variances of species data and eigenvalues are 3.58, 12.6 and 0.61 for the first, and 3.84, 21.1 and 0.41 for the second axis, respectively). The most frequent 30 species were added by weighted averaging; species were denoted using an eight-letter code with four letters of genus and four letters of species name. The average coordinates of the four subplots per plot were shown, numbers in boxes denotes fenced, while numbers without boxes the grazed plots. Notations: Site-1: 1–8, Site-2: 9–16, Site-3: 17–24.

**Figure 4:**
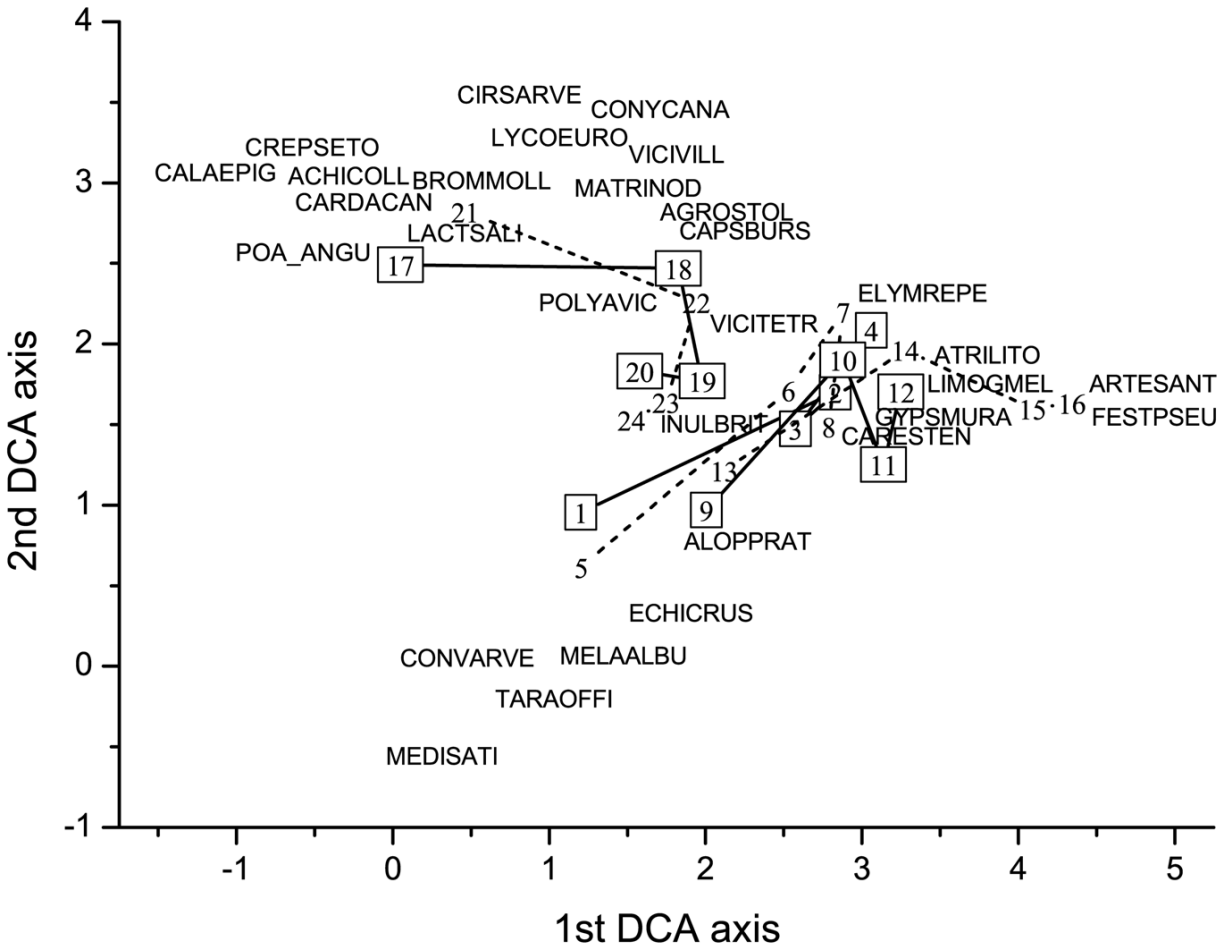
Vegetation changes in the mesophilous grasslands in the four years of the study. DCA ordination based on cover scores (gradient length, cumulative percentage variance of species data and eigenvalues are 4.59, 18.2, and 0.69 for the first, and 3.11, 29.2 and 0.42 for the second axis, respectively). For notations see Figure 2.

**Figure 5:**
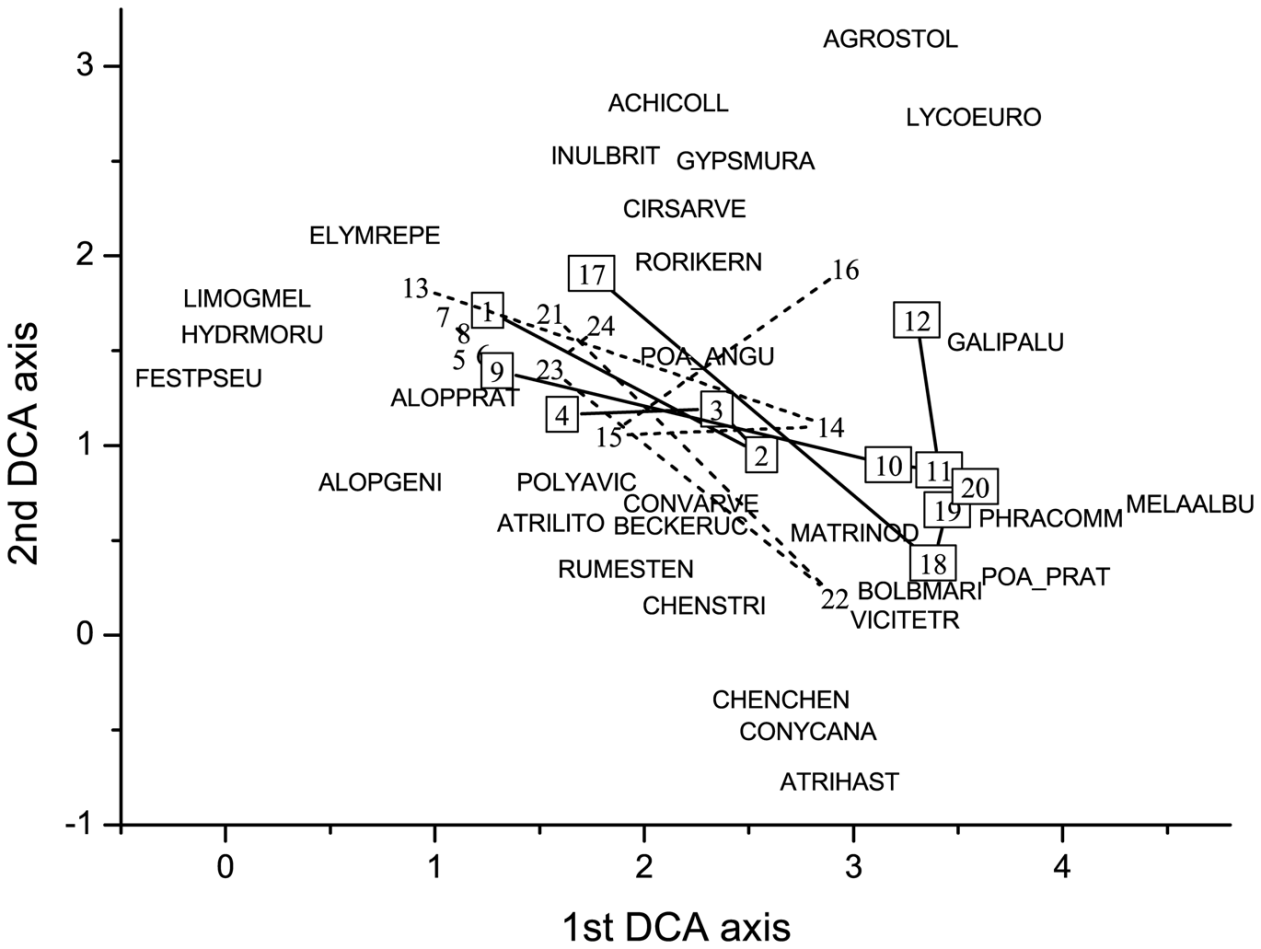
Vegetation changes in the wet grasslands in the four years of the study. DCA ordination based on cover scores (gradient length, cumulative percentage variance of species data and eigenvalues are 3.54, 20.1 and 0.75 for the first, and 2.89, 30.9 and 0.40 for the second axis, respectively). For notations see Figure 2.

More directional changes were detected in species composition of dry than in the other two grassland types. A convergent vegetation development was detected in grazed plots of secondary dry grasslands as was shown by the DCA ordination. The grazed sites regardless to their initial vegetation composition became more similar to each other than to their fenced counterparts (Figure 3). It is caused mostly by the cover increase of short grasses like *Festuca pseudovina* and *Poa angustifolia* (Figure 3). Conversely, the vegetation development in fenced plots was somewhat divergent (Figure 3). It is clearly shown that this was due by the uneven pattern and high cover of noxious species. For the other grassland types such clear trends were not detected. Changes of species composition in mesophilous grasslands were highly affected by the sites (as shown by the GLM analysis, Table 1): the grazed and fenced counterparts in a respective site were in the last year more similar to each other than to the plots of same management type in a different site (Figure 4). In the wet grassland type, high fluctuations occurred in the species composition both between years and sites, which were in line with the found numerous significant interaction effects in the GLM (Figure 5, Table 1).

## Discussion

### Effects of Grazing on Species Composition

It was formerly stressed that for conservation purposes indigenous breeds should be used because they are (i) more resistant to local weather extremities, parasites and diseases, (ii) they have the ability of utilising low-quality food sources and (iii) show a good reproductive performance [3]. Our results clearly demonstrated that grazing by Hungarian Grey cattle strongly affected the species richness and composition even in the short run. We also detected a remarkable effect on the specific heights: in all studied grassland types the cover-weighted specific heights were significantly lower in grazed plots. This was due to grazing benefitted creeping and rosette-forming species like *Plantago lanceolata, P. major, P. media, Taraxacum officinale, Trifolium repens* and *Polygonum aviculare*. Similar findings were reported in [18] for *Plantago lanceolata* and *Trifolium repens,* in [34] for *Plantago coronopus* and *Trifolium fragiferum*.

Suppression of tall-growing species was feasible in all studied grassland types; grazing selectively suppressed some tall-growing competitor species like *Elymus repens* or *Phragmites communis*. These findings are also in line with the findings of [18], where *Solidago gigantea* and *Arrhenatherum elatius* was suppressed by year-round grazing of Heck cattle. It was also found that cattle generally prefer sites with tall-growing vegetation and higher productivity, because it is much easier to obtain for cattle than much lower vegetation [3], [18]. Thus, in ungrazed sites generally tall-growing species occur [35]. Conversely, short-grasses like *Festuca pseudovina* or *Poa angustifolia* benefitted from grazing. These results were also supported by [36], where beneficial effects of cattle grazing were detected for the perennial short-grass *Danthonia californica*; or in the study of [37] for several annual short grasses.

### Diversity and Suppression of Noxious Species

It was formerly found that extensive cattle grazing has a positive effect on species diversity [3]. The positive effect on species richness is generally explained by the (i) lower diet selectivity of cattle compared to other foraging domestic livestock (i.e. species are not likely eliminated completely from the vegetation by cattle, [17]), (ii) opening spaces for less competitive species by the suppression of tall-growing dominant competitors [34], (iii) increased spatial heterogeneity and patchiness after cattle grazing [38] and by (iv) cattle-mediated seed dispersal [11]. In our study, significantly higher diversity of grazed plots was detected compared to fenced ones in mesophilous and wet grassland types.

Our results suggest that the traditional grazing by Hungarian Grey cattle can have beneficial effects already in the short run by the suppression of noxious species in all studied grassland types. The increase in biodiversity in mesophilous and wet grasslands was likely caused by the high rate of suppression of tall-growing noxious competitor species *Elymus repens* and *Phragmites communis*. These results were also supported by [34] for *Elymus repens*. It was also found that cattle grazing suppresses the biomass and reproductive success of *Phragmites communis* in grazed tall-herb fen vegetation (light grazing with 0.5–1 cattle/ha, [12]) and in seashore meadows (light grazing with 0.3–1.7 cattle/ha [39]). It was also reported by [40] that cattle grazing can transform a tall *Phragmites communis* dominated sward to a more heterogeneous vegetation with both tall and short species (0.25 cattle/ha, year round grazing).

The cover of short-lived noxious species was only considerable in the secondary dry grasslands, and this species group was effectively suppressed by grazing. Grazing was a feasible tool for suppressing thistle species, such as *Carduus acanthoides*. Short-lived weedy species are generally not considered as problem plants in grassland management, because they need regularly open spaces in grasslands for their establishment and recovery [41]. In contrast, it was stressed by [27], that short-lived noxious species can be present in high cover in the first years in the vegetation of grasslands restored by seed sowing, and their suppression by mowing in the first years can be costly (see also [1]). Our results clearly suggest that these species can be suppressed by Hungarian Grey cattle grazing.

### Specific Patterns of Grazing in Different Grassland Types

It was suggested by several studies that effects of grazing should be analysed in respect of the studied grassland types [36]. We found that, considering the same stocking rates (1 cattle/ha), the effect of grazing was quite different in grassland types along the dry-mesophilous-wet gradient. The effect of grazing was the most expressed in the wet grassland type; but yearly fluctuations were also the highest here. This is well in accordance with the findings of [42]: differences in yearly precipitation were stressed as an important masking factor for evaluation of grazing effects. Mostly moderate yearly fluctuations were detected in the dry grassland type, but compositional changes were more directed and not likely influenced by year-to-year differences (i.e. by precipitation differences). This was also demonstrated by the multivariate analyses (Figure 3). It was found in other studies that cattle grazing is not selective for most of the species, but a clear selectivity was found considering the feeding habitats [18], [34]. Thus, the detected differences between grassland types can also be explained by the higher selectivity of cattle for grasslands of higher vegetation height (i.e. for the mesophilous and wet grasslands compared to the dry ones). We can conclude that extensive Hungarian Grey cattle grazing is effective to suppress noxious species and to create a mosaic vegetation structure of short- and tall species in the short run, which enables to maintain high species richness in the landscape. In addition, Hungarian Grey cattle can feed in open habitats along long moisture gradient including also alkali marshes, thus, in highly mosaic landscapes it is better suited for grazing than other livestock types, which need a more homogeneous vegetation structure.

## Supporting Information

Table S1 **List of noxious species.** Perennial species were marked using boldface.
(DOC)

Table S2 **Vegetation characteristics of the studied grasslands.** Scores (mean±SD) were calculated based on the subplot scores for the secondary dry grasslands (A), mesophilous grasslands (B) and wet grasslands (C). For ‘noxious perennials’ and ‘noxious short-lived’ species the cover scores, for soil moisture (‘WB’) and ‘specific height’, cover-weighted scores were calculated and tested.
(DOC)
