# Supplementary material for: Traditional Cattle Grazing in a Mosaic Alkali Landscape: Effects on Grassland Biodiversity along a Moisture Gradient
Source: PLoS One. 2014 May 8;9(5):e97095. doi: 10.1371/journal.pone.0097095 (PMC4014582; doi:10.1371/journal.pone.0097095)
Supplement: Table S1 — List of noxious species. Perennial species were marked using boldface. (DOC) [file pone.0097095.s001.doc]

**Table S1.** **List of noxious species.** Perennial species were marked using boldface.

| *Amaranthus retroflexus* |
| --- |
| *Anagallis arvensis* |
| *Bilderdykia convolvulus* |
| *Bromus arvensis* |
| *Bromus mollis* |
| *Bromus tectorum* |
| ***Calamagrostis epigeios*** |
| *Capsella bursa-pastoris* |
| *Carduus acanthoides* |
| *Chenopodium album* |
| ***Cichorium intybus*** |
| ***Cirsium arvense*** |
| *Cirsium vulgare* |
| *Consolida regalis* |
| ***Covolvulus arvensis*** |
| *Conyza canadensis* |
| *Crepis setosa* |
| *Crepis tectorum* |
| *Cynoglossum officinale* |
| *Digitaria sanguinalis* |
| *Echinochloa crus-galli* |
| ***Elymus repens*** |
| *Hordeum murinum* |
| *Hyosciamus niger* |
| *Lactuca serriola* |
| *Lamium purpureum* |
| *Lepidium campestre* |
| ***Lolium perenne*** |
| *Malva neglecta* |
| *Matricaria inodora* |
| *Melandrium album* |
| ***Phragmites communis*** |
| *Picris hieracioides* |
| *Polygonum aviculare* |
| *Portulaca oleracea* |
| ***Rumex obtusifolius*** |
| ***Rumex patientia*** |
| *Setaria glauca* |
| *Setaria viridis* |
| *Solanum nigrum* |
| *Sonchus asper* |
| *Sonchus oleraceus* |
| *Stellaria media* |
| *Stenactis annua* |
| ***Taraxacum officinale*** |
| *Veronica persica* |
| *Vicia villosa* |
