# Supplementary material for: Traditional Cattle Grazing in a Mosaic Alkali Landscape: Effects on Grassland Biodiversity along a Moisture Gradient
Source: PLoS One. 2014 May 8;9(5):e97095. doi: 10.1371/journal.pone.0097095 (PMC4014582; doi:10.1371/journal.pone.0097095)
Supplement: Table S2 — Vegetation characteristics of the studied grasslands. Scores (mean±SD) were calculated based on the subplot scores for the secondary dry grasslands (A), mesophilous grasslands (B) and wet grasslands (C). For ‘noxious perennials’ and ‘noxious short-lived’ species the cover scores, for soil moisture (‘WB’) and ‘specific height’, cover-weighted scores were calculated and tested. (DOC) [file pone.0097095.s002.doc]

**Table S2. Vegetation characteristics of the studied grasslands.**

| **A** |  |  | **Shannon diversity** | **Species richness** | **Specific height** | **Soil moisture (WB)** | **Noxious perennials** | **Noxious short-lived** |
| --- | --- | --- | --- | --- | --- | --- | --- | --- |
| **Site 1** | **Fenced** | 2006 | 1.32 ± 0.15 | 13.25 ± 3.10 | 34.88 ± 7.72 | 4.79 ± 0.31 | 70.90 ± 21.39 | 5.65 ± 3.18 |
|  |  | 2007 | 1.66 ± 0.18 | 10.00 ± 1.41 | 42.77 ± 8.14 | 4.58 ± 0.40 | 56.33 ± 9.66 | 9.65 ± 9.80 |
|  |  | 2008 | 1.23 ± 0.14 | 9.25 ± 3.20 | 55.29 ± 8.71 | 4.53 ± 0.50 | 31.20 ± 22.79 | 5.13 ± 3.25 |
|  |  | 2009 | 1.50 ± 0.32 | 13.25 ± 2.75 | 48.49 ± 12.82 | 3.93 ± 0.51 | 48.60 ± 21.14 | 9.95 ± 6.06 |
|  | **Grazed** | 2006 | 1.41 ± 0.39 | 12.00 ± 1.15 | 32.82 ± 4.40 | 4.63 ± 0.31 | 64.73 ± 30.24 | 5.15 ± 3.07 |
|  |  | 2007 | 1.41 ± 0.52 | 8.25 ± 2.63 | 43.21 ± 11.37 | 4.54 ± 0.34 | 76.85 ± 16.35 | 1.43 ± 1.39 |
|  |  | 2008 | 1.75 ± 0.26 | 11.25 ± 1.50 | 42.37 ± 9.52 | 4.10 ± 0.44 | 39.55 ± 12.77 | 15.4 ± 21.85 |
|  |  | 2009 | 1.62 ± 0.37 | 13.25 ± 4.03 | 42.29 ± 9.63 | 3.65 ± 0.33 | 14.23 ± 6.92 | 3.28 ± 2.45 |
| **Site 2** | **Fenced** | 2006 | 1.51 ± 0.32 | 10.50 ± 2.65 | 48.02 ± 6.55 | 4.82 ± 0.39 | 48.40 ± 19.45 | 8.95 ± 9.78 |
|  |  | 2007 | 1.72 ± 0.11 | 12.75 ± 1.50 | 36.83 ± 6.37 | 3.81 ± 0.36 | 37.58 ± 17.93 | 9.10 ± 4.44 |
|  |  | 2008 | 1.15 ± 0.12 | 9.50 ± 1.00 | 43.48 ± 3.33 | 3.16 ± 0.62 | 1.45 ± 0.77 | 40.68 ± 39.02 |
|  |  | 2009 | 1.69 ± 0.20 | 10.00 ± 1.63 | 41.97 ± 0.76 | 3.69 ± 0.38 | 5.73 ± 5.30 | 41.13 ± 8.36 |
|  | **Grazed** | 2006 | 1.56 ± 0.13 | 13.00 ± 2.45 | 37.99 ± 4.30 | 4.36 ± 0.46 | 58.43 ± 16.01 | 3.23 ± 1.54 |
|  |  | 2007 | 1.36 ± 0.43 | 11.75 ± 4.03 | 37.55 ± 1.81 | 3.69 ± 0.66 | 30.60 ± 24.04 | 13.05 ± 7.29 |
|  |  | 2008 | 1.29 ± 0.46 | 10.75 ± 3.10 | 41.83 ± 4.89 | 3.93 ± 0.61 | 12.28 ± 7.79 | 2.13 ± 1.00 |
|  |  | 2009 | 1.31 ± 0.49 | 10.50 ± 2.38 | 36.20 ± 1.51 | 3.25 ± 0.13 | 14.45 ± 15.96 | 3.03 ± 3.37 |
| **Site 3** | **Fenced** | 2006 | 1.63 ± 0.19 | 12.50 ± 1.29 | 66.85 ± 6.51 | 3.66 ± 0.26 | 33.05 ± 15.38 | 54.10 ± 14.28 |
|  |  | 2007 | 1.88 ± 0.18 | 16.25 ± 1.50 | 60.27 ± 7.13 | 3.37 ± 0.37 | 14.40 ± 12.53 | 52.35 ± 10.78 |
|  |  | 2008 | 1.39 ± 0.33 | 12.50 ± 2.38 | 65.49 ± 11.69 | 3.17 ± 0.10 | 2.23 ± 1.09 | 59.63 ± 17.33 |
|  |  | 2009 | 1.60 ± 0.24 | 11.75 ± 1.71 | 54.63 ± 12.97 | 2.96 ± 0.25 | 0.80 ± 0.75 | 41.23 ± 26.07 |
|  | **Grazed** | 2006 | 1.49 ± 0.21 | 12.25 ± 3.10 | 55.20 ± 1.51 | 4.08 ± 0.44 | 20.28 ± 5.90 | 47.50 ± 26.01 |
|  |  | 2007 | 1.80 ± 0.39 | 16.00 ± 2.31 | 55.01 ± 7.54 | 3.70 ± 0.45 | 31.53 ± 24.61 | 27.05 ± 20.74 |
|  |  | 2008 | 1.47 ± 0.27 | 13.25 ± 1.50 | 55.71 ± 19.62 | 3.65 ± 0.48 | 30.75 ± 27.46 | 5.75 ± 3.11 |
|  |  | 2009 | 1.79 ± 0.22 | 14.75 ± 2.63 | 52.36 ± 15.64 | 3.47 ± 0.71 | 18.88 ± 20.97 | 11.35 ± 5.39 |

| **B** |  |  | **Shannon diversity** | **Species richness** | **Specific height** | **Soil moisture (WB)** | **Noxious perennials** | **Noxious short-lived** |
| --- | --- | --- | --- | --- | --- | --- | --- | --- |
| **Site 1** | **Fenced** | 2006 | 1.30 ± 0.30 | 8.00 ± 0.82 | 50.01 ± 9.84 | 4.90 ± 0.54 | 62.04 ± 29.58 | 4.30 ± 3.78 |
|  |  | 2007 | 0.56 ± 0.22 | 4.25 ± 1.50 | 72.33 ± 1.38 | 5.22 ± 0.14 | 78.26 ± 14.71 | 0.00 ± 0.00 |
|  |  | 2008 | 0.77 ± 0.11 | 4.50 ± 1.29 | 70.67 ± 1.15 | 5.23 ± 0.07 | 68.49 ± 3.69 | 0.20 ± 0.23 |
|  |  | 2009 | 0.13 ± 0.15 | 2.75 ± 1.26 | 74.03 ± 1.18 | 5.02 ± 0.03 | 97.25 ± 3.39 | 0.03 ± 0.06 |
|  | **Grazed** | 2006 | 1.25 ± 0.24 | 8.00 ± 2.45 | 47.00 ± 7.08 | 5.12 ± 0.60 | 50.33 ± 16.97 | 1.32 ± 1.64 |
|  |  | 2007 | 0.79 ± 0.22 | 5.00 ± 0.00 | 69.53 ± 2.68 | 5.33 ± 0.23 | 64.16 ± 25.18 | 1.29 ± 1.49 |
|  |  | 2008 | 0.66 ± 0.15 | 6.50 ± 2.65 | 70.35 ± 1.74 | 5.10 ± 0.10 | 82.38 ± 7.38 | 0.86 ± 1.72 |
|  |  | 2009 | 1.48 ± 0.17 | 8.50 ± 2.65 | 48.80 ± 6.40 | 5.28 ± 0.23 | 44.56 ± 8.77 | 1.55 ± 1.10 |
| **Site 2** | **Fenced** | 2006 | 0.44 ± 0.14 | 3.25 ± 0.96 | 63.98 ± 58.38 | 6.15 ± 0.10 | 0.56 ± 0.38 | 8.33 ± 10.93 |
|  |  | 2007 | 1.40 ± 0.26 | 8.25 ± 2.06 | 57.87 ± 4.72 | 4.62 ± 0.31 | 35.45 ± 18.72 | 22.17 ± 23.96 |
|  |  | 2008 | 1.25 ± 0.32 | 6.75 ± 1.26 | 61.04 ± 3.66 | 5.19 ± 0.58 | 30.26 ± 10.65 | 0.13 ± 0.27 |
|  |  | 2009 | 1.12 ± 0.53 | 6.25 ± 1.71 | 62.71 ± 7.28 | 4.79 ± 0.20 | 57.64 ± 25.40 | 0.37 ± 0.45 |
|  | **Grazed** | 2006 | 1.04 ± 0.59 | 5.00 ± 2.16 | 58.38 ± 4.57 | 5.96 ± 0.46 | 27. 31 ± 39.20 | 3.63 ± 4.14 |
|  |  | 2007 | 1.11 ± 0.44 | 9.50 ± 1.29 | 64.43 ± 5.84 | 4.88 ± 0.09 | 67.71 ± 22.17 | 1.98 ± 1.40 |
|  |  | 2008 | 1.18 ± 0.30 | 6.50 ± 1.29 | 44.67 ± 7.42 | 3.85 ± 0.74 | 14.04 ± 18.00 | 0.07 ± 0.15 |
|  |  | 2009 | 1.10 ± 0.07 | 7.00 ± 1.15 | 39.38 ± 0.87 | 3.58 ± 0.36 | 6.57 ± 4.98 | 0.29 ± 0.34 |
| **Site 3** | **Fenced** | 2006 | 1.13 ± 0.38 | 11.25 ± 1.71 | 43.93 ± 4.31 | 3.17 ± 0.07 | 6.22 ± 5.53 | 20.34 ± 2.21 |
|  |  | 2007 | 1.71 ± 0.10 | 10.50 ± 1.00 | 65.52 ± 3.19 | 4.61 ± 0.39 | 35.35 ± 9.46 | 37.10 ± 6.86 |
|  |  | 2008 | 1.24 ± 0.22 | 9.00 ± 2.94 | 63.45 ± 8.11 | 5.11 ± 0.56 | 33.31 ± 25.39 | 5.01 ± 6.03 |
|  |  | 2009 | 1.22 ± 0.26 | 9.00 ± 1.41 | 62.91 ± 8.27 | 4.77 ± 0.73 | 41.82 ± 17.95 | 3.85 ± 2.75 |
|  | **Grazed** | 2006 | 1.70 ± 0.19 | 13.50 ± 2.52 | 55.30 ± 10.37 | 3.78 ± 0.18 | 37.41 ± 22.05 | 26.22 ± 16.62 |
|  |  | 2007 | 1.68 ± 0.15 | 12.75 ± 1.71 | 65.60 ± 3.18 | 4.89 ± 0.12 | 36.94 ± 20.12 | 32.93 ± 10.04 |
|  |  | 2008 | 1.27 ± 0.14 | 13.00 ± 0.82 | 61.50 ± 4.48 | 5.14 ± 0.35 | 22.87 ± 23.20 | 3.28 ± 1.60 |
|  |  | 2009 | 1.26 ± 0.17 | 11.75 ± 1.26 | 57.72 ± 6.30 | 4.98 ± 0.51 | 12.81 ± 7.19 | 2.53 ± 0.90 |

| **C** |  |  | **Shannon diversity** | **Species richness** | **Specific height** | **Soil moisture (WB)** | **Noxious perennials** | **Noxious short-lived** |
| --- | --- | --- | --- | --- | --- | --- | --- | --- |
| **Site 1** | **Fenced** | 2006 | 0.53 ± 0.35 | 5.50 ± 0.58 | 57.66 ± 7.43 | 6.75 ± 0.81 | 1.54 ± 0.71 | 0.00 ± 0.00 |
|  |  | 2007 | 0.90 ± 0.16 | 6.50 ± 1.73 | 135.36 ± 34.81 | 8.05 ± 1.04 | 54.37 ± 26.03 | 0.32 ± 0.63 |
|  |  | 2008 | 1.09 ± 0.26 | 7.75 ± 0.96 | 130.24 ± 42.01 | 7.78 ± 1.28 | 61.70 ± 21.08 | 5.27 ± 6.19 |
|  |  | 2009 | 1.30 ± 0.25 | 6.00 ± 2.16 | 87.35 ± 23.57 | 6.30 ± 0.91 | 38.88 ± 15.96 | 0.39 ± 045 |
|  | **Grazed** | 2006 | 0.50 ± 0.27 | 5.00 ± 0.82 | 54.65 ± 9.89 | 7.15 ± 1.10 | 0.64 ± 0.07 | 0.00 ± 0.00 |
|  |  | 2007 | 0.53 ± 0.16 | 6.50 ± 1.73 | 66.51 ± 1.62 | 5.93 ± 0.08 | 11.45 ± 7.71 | 0.37 ± 0.48 |
|  |  | 2008 | 0.80 ± 0.13 | 4.75 ± 1.71 | 67.98 ± 1.27 | 5.57 ± 0.12 | 38.10 ± 10.24 | 0.42 ± 0.40 |
|  |  | 2009 | 0.91 ± 0.15 | 5.00 ± 0.82 | 67.01 ± 1.75 | 5.70 ± 0.08 | 29.02 ± 3.31 | 1.79 ± 1.14 |
| **Site 2** | **Fenced** | 2006 | 0.64 ± 0.23 | 4.75 ± 2.87 | 63.33 ± 1.18 | 5.91 ± 5.41 | 8.18 ± 4.29 | 0.10 ± 0.20 |
|  |  | 2007 | 0.71 ± 0.50 | 6.50 ± 1.73 | 170.31 ± 25.43 | 9.31 ± 0.72 | 79.00 ± 17.91 | 1.82 ± 2.89 |
|  |  | 2008 | 0.64 ± 0.44 | 7.00 ± 2.58 | 176.29 ± 19.71 | 9.46 ± 0.48 | 83.51 ± 12.96 | 1.24 ± 1.03 |
|  |  | 2009 | 0.99 ± 0.25 | 7.00 ± 1.15 | 126.46 ± 32.31 | 8.69 ± 0.69 | 52.76 ± 20.25 | 0.41 ± 0.53 |
|  | **Grazed** | 2006 | 0.63 ± 0.30 | 3.75 ± 1.26 | 70.04 ± 4.69 | 5.41 ± 0.30 | 58. 61 ± 32.51 | 0.00 ± 0.00 |
|  |  | 2007 | 1.41 ± 0.42 | 11.75 ± 4.35 | 135.55 ± 28.84 | 8.19 ± 1.07 | 61.32 ± 13.33 | 3.00 ± 1.48 |
|  |  | 2008 | 0.68 ± 0.24 | 9.00 ± 3.16 | 106.28 ± 76.24 | 6.32 ± 3.42 | 44.05 ± 43.61 | 3.02 ± 4.44 |
|  |  | 2009 | 1.48 ± 0.19 | 13.00 ± 2.58 | 85.09 ± 15.48 | 6.96 ± 1.13 | 32.57 ± 15.43 | 1.53 ± 1.45 |
| **Site 3** | **Fenced** | 2006 | 0.54 ± 0.07 | 3.00 ± 0.82 | 62.45 ± 11.45 | 5.94 ± 0.54 | 15.67 ± 9.29 | 0.00 ± 0.00 |
|  |  | 2007 | 0.66 ± 0.21 | 4.75 ± 1.71 | 168.4 ± 10.56 | 9.25 ± 0.22 | 77.52 ± 7.73 | 0.07 ± 0.14 |
|  |  | 2008 | 0.43 ± 0.08 | 4.50 ± 1.29 | 185.34 ± 6.36 | 9.58 ± 0.30 | 90.51 ± 4.43 | 4.51 ± 6.65 |
|  |  | 2009 | 0.34 ± 0.20 | 4.25 ± 0.50 | 189.04 ± 8.19 | 9.70 ± 0.32 | 93.24 ± 6.24 | 2.73 ± 4.81 |
|  | **Grazed** | 2006 | 0.73 ± 0.27 | 3.75 ± 0.96 | 76.63 ± 8.56 | 5.47 ± 078 | 43.07 ± 29.60 | 0.09 ± 0.19 |
|  |  | 2007 | 1.73 ± 0.09 | 13.25 ± 1.89 | 102.4 ± 8.19 | 7.25 ± 0.25 | 35.92 ± 9.51 | 5.67 ± 0.78 |
|  |  | 2008 | 1.54 ± 0.18 | 10.50 ± 2.08 | 74.61 ± 11.11 | 5.66 ± 0.72 | 32.11 ± 7.84 | 3.55 ± 4.45 |
|  |  | 2009 | 1.61 ± 0.23 | 10.50 ± 2.38 | 63.61 ± 13.80 | 5.75 ± 0.61 | 18.56 ± 14.04 | 2.66 ± 1.96 |

Scores (mean±SD) were calculated based on the subplot scores for the secondary dry grasslands (A), mesophilous grasslands (B) and wet grasslands (C). For ‘noxious perennials’ and ‘noxious short-lived’ species the cover scores, for soil moisture (‘WB’) and ‘specific height’ cover-weighted scores were calculated and tested.
